# Single-Walled and Multiwalled Carbon Nanotubes in Polybutadiene/Natural Rubber Composites Containing Silica for Conductive Green-Tire Application

**DOI:** 10.1021/acsomega.5c01638

**Published:** 2025-07-01

**Authors:** Priscila Almeida Lucio Campini, Felipe Gustavo Ornaghi, Marcus Vinicius Braum, Elyff Cargnin, Guilherme Barnez Gramcianinov, Diego Moreira Lima, Renata dos Santos Pereira, Demétrio Jackson dos Santos, Anne Cristine Chinellato, Suel Eric Vidotti, Danilo Justino Carastan, Mathilde Champeau

**Affiliations:** † Center of Engineering, Modeling and Applied Social Sciences, Federal University of ABC, Santo André, SP 09210-580, Brazil; ‡ PROMETEON TYRE GROUP, Av. Alexandre de Gusmão, 487 - Vila Homero Thon, Santo André, SP 09111-310, Brazil

## Abstract

This study investigates the influence of commercial single-walled (SWCNT) and multiwalled carbon nanotubes (MWCNT) on the properties of natural rubber/polybutadiene composites containing silica for green tire applications. Composites were prepared on a laboratory scale using mixing protocols simulating conventional industrial methods, varying the nanotube content while keeping a fixed silica concentration of 55 phr. First, SWCNT and MWCNT were characterized in terms of atomic composition, number of defects, and morphology. Electrical conductivity measurements on nanocomposites indicate that SWCNT achieves the percolation threshold at lower concentrations (between 8 and 10 phr) compared to MWCNT (between 10 and 13.5 phr). Electrical conductivity in the range of antistatic properties (10^–4^ to 10^–8^ S·m^–1^) is achieved at 10 and 13.5 phr in both cases. Differences in nanotube dispersion were observed, with SWCNT forming long bundles and MWCNT exhibiting more entangled structures. Dynamic mechanical analysis shows that SWCNT increases the storage modulus and reduces the glass transition peak width more effectively than MWCNT, indicating superior reinforcement and reduced polymer mobility. Considering the sample with good dispersion and desired electrical conductivity (10 phr SWCNT and 13.5 phr MWCNT), dynamic mechanical analysis indicated that MWCNT leads to lower values of rolling resistance than SWCNT, while wet grip improves with the addition of SWCNT. The study concludes that SWCNT provides a more efficient conductive pathway and reinforcing effect than MWCNT at equivalent loadings, offering insights into optimizing conductive elastomer composites for energy-efficient tire technologies.

## Graphical abstract

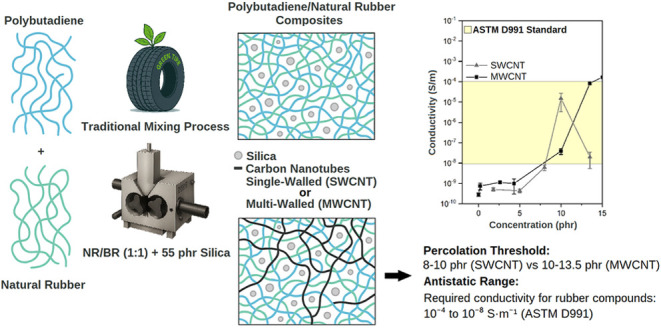

## 1 Introduction

According to the International Energy Agency (IEA), the global transport sector accounted for 25% of the total energy consumed in 2020.^1^ A proposed strategy to mitigate energy consumption is the reduction of average fuel consumption in vehicles. Tires, particularly tire treads, are the direct point of contact with the ground and play a pivotal role in fuel consumption reduction. New technologies are necessary to enhance tire energy efficiency, primarily by minimizing tire tread rolling resistance, which is responsible for 20–30% of vehicle fuel consumption.^2,3^ The key factor contributing to rolling resistance is hysteresis, a phenomenon arising from cyclic deformation during vehicle movement, leading to internal energy losses. Hysteresis is attributed to friction between reinforcing particles in the elastomeric matrix as well as due to the viscoelastic properties of polymers, accounting for 80–95% of rolling resistance.^4^ In addition to low rolling resistance, the tire tread should maintain high abrasion resistance, offer excellent wet grip, ensure durability, and enhance safety.

Historically, carbon black has been the reinforcing filler in tire treads. However, the advent of ’green tires’ in the 1990s proposed substituting carbon black with silica modified by organosilane to reduce hysteresis.^5,6^ Green tires typically contain from 20 to 60 phr of silica.^7–10^ While the substitution of carbon black for silica decreases hysteresis, it introduces challenges such as static charge generation during tire rolling due to the insulating nature of silica particles. The ASTM D991 standard establishes the required range of electrical resistivity for antistatic rubber products between 10^4^ to 10^8^ Ω·m, which corresponds to electrical conductivities in the range of 10^–4^ to 10^–8^ S·m^–1^.^11^ Thus, to counterbalance the loss of electrical conductivity in silica-filled rubber, the addition of conductive particles in the formulation has been proposed to dissipate static energy.^12^ Carbon nanotubes (CNTs) have garnered significant interest among the various conductive nanoparticles. Their potential to establish conductive pathways within the elastomer matrix, coupled with their dual role as reinforcing fillers, makes them particularly promising. This is attributed to their high electrical conductivity, high aspect ratio, and impressive elastic modulus.^13–16^

CNTs can be categorized into single-walled (SWCNT), consisting of a monolayer with a diameter of less than 2 nm, and multiwalled (MWCNT), which can have 6–50 concentric layers. While SWCNTs promote higher electrical conductivity, MWCNTs are a cost-effective alternative due to their ease of production and superior dispersion, making them more prevalent in practical applications.^13–16^

Electrical conductivity of CNT-filled nanocomposites depends on shape, size, aspect ratio, and dispersion of the CNTs as well as the polymer-CNT interaction. The dispersion of these nanofillers is particularly difficult due to their substantial van der Waals interactions, π-π stacking between nanotubes, and their high aspect ratio, which leads to the formation of entangled agglomerates.^6,12,17–19^ Achieving the desired electrical conductivity in the nanocomposite while minimizing the quantity of fillers depends on their adequate dispersion in the matrix. Traditional elastomer processing technologies, such as internal mixers and roll-milling, make this dispersion particularly difficult.^20,21^

Previous studies have proposed MWCNT nanotube modification prior to incorporation in elastomer compounds to facilitate mixing.^22–27^ Moreover, a few other studies have investigated the introduction of MWCNT in elastomer compounds containing silica prepared by melt-mixing processes.^28–34^ Fritzsche et al. incorporated different silica/MWCNT ratios in natural rubber (NR) containing high silica content (80 to 90 phr) by melt-mixing and achieved a high conductivity value (10^–3^ S·cm^–1^) with 6 phr of MWCNT (0.6 vol %).^29^ Park et al.^31^ investigated the impact of the addition of MWCNT with three different lengths (5, 30, and 100 μm) and three different quantities (1, 2, 4, and 7 phr) on the properties of styrene butadiene rubber (SBR) compounds containing 80 phr of silica. Incorporating 4 phr of 30 and 100 μm-length MWCNT enabled it to pass the percolation threshold, reaching 4 × 10^–8^ S·cm^–1^, and 7 phr led to 2 × 10^–5^ S·cm^–1^. However, it negatively affected hysteresis, rolling resistance, and abrasion of these compounds.^31^ Another work developed hybrid SiO_2_/MWCNT nanoparticles to improve both mechanical and electrical properties, but the application of such particles is limited because they are not commercially available.^35^

Regarding studies focused on rubber compounds with silica and SWCNT, they are only available for lower silica contents. Bakošová and Bakošová added only 8 phr of silica in an elastomer compound composed of NR/butadiene/isoprene/styrene–butadiene, containing from 1 to 2 phr of SWCNT.^28^ In addition, most of these studies do not compare the performance of elastomer compounds containing SWCNTs and MWCNTs.^36–38^ To address the scarcity of comparative studies, Kumar and Lee investigated SWCNTs and MWCNTs as reinforcing agents in silicone rubber, evaluating dispersion, tensile and compressive properties, and electrical resistance in formulations with up to 3 phr of CNTs. However, CNTs dispersion in silicone was performed manually, and the viscoelastic properties of silicone are distinct from the elastomers used in tire treads.^36^ In another work, Adu et al. (under review) prepared different nanocomposites of NBR containing either SW or MWCNT in different proportions (0.56–7.05 vol %), by mixing the dispersion of CNT in NBR latex using a magnetic stirrer, which is a wet state technique with low dispersion capacity.^39^

Building upon this groundwork, our study aims to compare the performance of silica-filled elastomer compounds containing SWCNTs and MWCNTs. In our experimental design, natural rubber/polybutadiene (NR/BR 1:1) compounds were prepared with a fixed precipitated silica content (55 phr) and varying quantities of commercial SWCNTs and MWCNTs without previous modification. The NR/BR 1:1 ratio, the silica content, and the formulation correspond to a typical green-tire formulation for preparing heavy vehicle tire treads.^40–44^ We employed traditional mixing equipment — an internal mixer and a two-roll mill — which are both commonly used for preparing such compounds. The characterization phase encompassed an examination of the morphology, structure, and atomic composition of SWCNT and MWCNT. The addition of SWCNT between 0 and 13.5 phr and of MWCNT between 0 and 24 phr was investigated. CNT dispersion was meticulously observed using scanning electron microscopy, and percolation curves unveiled antistatic electrical properties. The impact of the addition of SWCNT and MWCNT on the vulcanization kinetics, as well as on dynamic properties, tensile properties, and hardness, was also investigated.

## 2 Materials and Methods

### 2.1 Materials

A blend of natural rubber (NR) and polybutadiene rubber (BR) (ratio 1:1) was used as the elastomeric matrix (Table 1). The system was reinforced with precipitated silica and cured with a sulfur-based vulcanization system. Other materials are listed in Table 1. Prometeon Tyres Group supplied all materials, which were utilized as received.

**Table 1:** Formulation of Rubber Compounds

ingredients	phr
NR	50
*Cis*-BR	50
highly dispersible precipitated silica	55
CNT	0–24
bis[3-(triethoxysilyl)propyl] tetrasulfide (TESPT)	5.5
protective agents	5.0
processing aids	8.0
zinc oxide	3.5
stearic acid	2.0
sulfenamide accelerator	1.3
sulfur	1.7

Two types of carbon nanotubes were used as nanofillers: multiwalled (MWCNT) (Nanoview, carbon purity >93%, external diameter 10–30 nm, length 5–30 μm, surface area >150 mg·m^2^/g, elementary composition: 96.6% carbon, 0.2% oxygen, impurities: Co, Fe, Al_2_O_3_) and single-walled (SWCNT) (Tuball by OCSiAl, carbon purity 87.1%, external diameter 1.6 ± 0.4 nm, average length 5 μm, surface area 300 mg·m^2^/g, elementary composition: > 85%, impurities 10%).

### 2.2 Processing of Rubber Compounds

Filler loading in composite systems varied from 0 to 13.5 phr of SWCNT, and from 0 to 24 phr of MWCNT, with a fixed concentration of precipitated silica (55 phr). A reference sample without CNT was also prepared, referred to as “reference”. Compositions were prepared in an internal mixer (Plastograph EC + Banbury type mixer W 50 ET, Brabender), at 80 rpm rotor speed, temperature of 80 °C, and 0.7 fill factor. The samples were further processed in a two-roll mill for cooling and sheet formation. The processing was carried out in three following stages, with a minimum interval of 30 min between stages:

Stage 1 (Internal Mixer): the rubbers (NR and BR) and fillers (55 phr silica, TESPT, and resins) were mixed in the internal mixer. This step ensured the initial incorporation of silica into the elastomer matrix.

Stage 2 (Internal Mixer): after at least 30 min, 0–24 phr CNT, rubber additives (processing aids, protective agents, and zinc oxide), and stearic acid were added to the internal mixer under the same temperature and mixing speed conditions. This stage allowed for uniform distribution of CNT.

Stage 3 (Internal Mixer and Two-Roll Mill): following another 30 min interval, sulfur and accelerators were added to the internal mixer. The compound was then transferred to the two-roll mill for cooling and sheet formation, finalizing the mixing and shaping of the samples. After 24h, the compounds were cured in a heated hydraulic press at 170 °C at their respective optimum cure time (*t*_90_).

### 2.3 Characterization of CNT

#### 2.3.1 Scanning Electron Microscopy

The structure and morphology of the nanotubes were observed by field emission scanning electron microscopy (FE-SEM) MIRA4 from TESCAN (Brno, The Czech Republic) at magnifications of 5000× and 200,000×. The nominal resolution of the system is approximately 2–5 nm. Nanoparticles were deposited onto a silicon oxide substrate adhered to carbon tape.

#### 2.3.2 X-ray Photoelectron Spectroscopy

The atomic composition of the nanoparticles was investigated by X-ray photoelectron spectroscopy (XPS). The SWCNT spectrum was obtained in K-Alpha+ (ThermoFisher Scientific), with Al Kα X-ray source (*h*ν= 1486,7 eV) and argon beams for charge compensation and penetration of 10 nm. The MWCNT spectrum was obtained in Scienta-Omicron ESCA, with Al Kα X-ray source, high-performance hemispherical analyzer, and low-energy source for charge compensation. Data processing was performed using CasaXPS software.

#### 2.3.3 Raman Spectroscopy

Raman spectroscopy for SWCNTs was performed in an Alpha300 spectrometer (Witec) using an incident laser with a wavelength of 532 nm (green light) and 5.0 mW laser power. Wet nanoparticles were dispersed in isopropyl alcohol using an ultrasonic bath, deposited onto a silicon wafer, and oven-dried for 90 min at 90 °C. In the case of MWCNT, tests were carried out in a T64000 Raman Spectrometer (Horija Jobin-Yvon), using an incident laser with a wavelength of 532 nm (green light), and laser power below 5.0 mW. The treatment for MWCNTs samples was the same as for SWCNT. Data processing was performed using Origin Pro 8.5, with D and G bands adjustment using a Lorentzian function.

### 2.4 Characterization of Rubber Compounds

#### 2.4.1 Scanning Electron Microscopy

The dispersion of silica and nanotubes in the NR/BR compounds was evaluated using a field emission scanning electron microscope (FE-SEM) MIRA4 from TESCAN (Brno, the Czech Republic). The material was cryo-fractured, and the fracture surface was analyzed after coating with a 5 nm gold layer deposited by sputtering. The reference sample, as well as the nanocomposites with 10 and 13.5 phr SWCNT and MWCNT, were analyzed.

#### 2.4.2 Electrical Properties

The electrical properties were measured with an SI 1260A gain phase analyzer coupled with a 1296A dielectric interface from Solartron (Leicester, United Kingdom). Dielectric spectra were taken from 0.1 Hz to 1 MHz with an applied AC voltage of 1 or 3 V, depending on the sample’s resistivity. AC conductivity was calculated from the imaginary permittivity at the lowest frequency (0.1 Hz) using the eq 1, where ω is the angular frequency, ε_0_ is the vacuum permittivity, and ε″ (ω) is the imaginary permittivity at the applied angular frequency. Results were averaged from five disc-shaped test specimens, each measuring 1 mm in thickness and 16 mm in diameter.

1 $$\sigma_{\mathrm{AC}}=\omega \varepsilon_{0}\varepsilon^{\prime \prime }\left(\omega \right)$$

Further characterizations were performed for the samples containing between 5 and 13.5 phr of SWCNT to evaluate the influence of nanofiller concentration on nanocomposite properties, considering that studies with SWCNT in elastomers are scarce. Samples with MWCNT that exhibited antistatic properties, i.e., with 10 and 13.5 phr, were also characterized. The comparison of nanocomposites with 10 and 13.5 phr of SWCNT and MWCNT permitted examining the influence of CNT on the properties of the tire tread compound.

#### 2.4.3 Vulcanization

The behavior of compounds during vulcanization was evaluated using a Rubber Process Analyzer RPA 2000 from α Technologies (Wiltshire, UK) at 170 °C for 10 min. Scorch time (*t*_s_), optimum cure time (*t*_90_), maximum (*M*_H_), and minimum (*M*_L_) torques were obtained as per the standard ASTM D5289 (Standard Test Method for Rubber Property—Vulcanization Using Rotorless Cure Meters) procedure. The samples with 5 to 13.5 phr of SWCNT, and with 10 and 13.5 phr were characterized to observe the influence of SWCNT concentration and to compare the properties of the samples with an electrical conductivity within the antistatic conductivity range.

#### 2.4.4 Payne Effect

The Payne effect was measured on vulcanized samples on the RPA 2000 equipment from α Technologies. The strain amplitude varied from 0 to 250%, at a frequency of 0.1 Hz. For the evaluation of the dispersion state of the fillers, a strain range between 0 and 28% was used.

#### 2.4.5 Rolling Resistance and Wet Adhesion

Rolling resistance and wet grip performance were investigated by dynamic mechanical analysis (DMA) through a temperature sweep from −80 to 100 °C, with a heating ramp of 3 °C/min, at a fixed frequency of 10 Hz, strain amplitude of 0.1% and preload force of 0.01 N, in N_2_ atmosphere (Q800 and Discovery DMA 850, TA Instruments). The results were an average of three test specimens.

#### 2.4.6 Tensile Test

Tensile tests were carried out according to ASTM D412 (Standard Test Methods for Vulcanized Rubber and Thermoplastic Elastomers—Tension), die C, using an EMIC DL 5000/10000 universal testing machine with a load cell of 200 N and a rate of 50 mm/min. The results were an average of five test specimens.

#### 2.4.7 Hardness Shore D

The sample hardness was determined using a durometer with a Shore D scale, according to ASTM D2240 Shore D (Standard Test Method for Rubber Property—Durometer Hardness). Results were averaged from eight measurements.

## 3 Results and Discussion

### 3.1 Characterization of SWCNT and MWCNT

#### 3.1.1 Scanning Electron Microscopy (SEM)

SEM micrographs (Figure 1) show that SWCNTs initially grow as individual tubes and subsequently aggregate into larger bundles to reduce surface energy.^45^ SWCNTs form fiber-like bundles of a few micrometers in length (Figure 1a), with no significant presence of metallic particles from synthesis catalysts (Figure 1b). Figure 1c reveals that these fibers are composed of SWCNT agglomerates with an average diameter of (40  ±  8) nm, suggesting that the individual nanotubes are even thinner. In contrast, MWCNTs are presented as entangled individual nanotubes without distinguishable clusters (Figure 1d), displaying a more curved and slender morphology (Figure 1e). Although their length cannot be measured from SEM images due to entanglements, the average diameter measured was (30  ±  6) nm (Figure 1f), which is consistent with the range provided by the supplier (10–30 nm).

**Figure 1:**
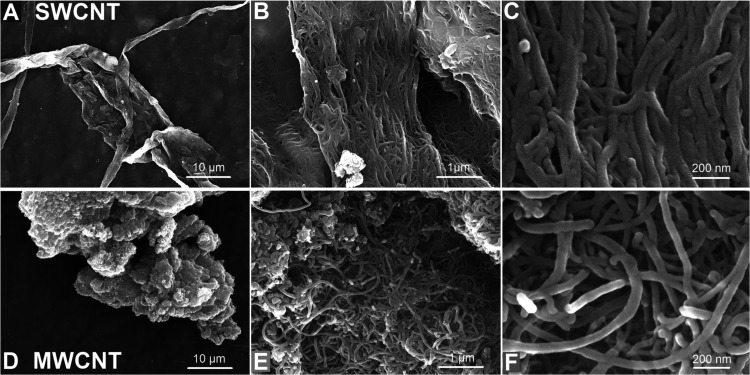
SEM micrographs of SWCNT (a, b, and c) and MWCNT (d, e, and f).

#### 3.1.2 Atomic Composition

The atomic composition of SWCNT and MWCNT was measured by XPS (Figure 2 and Table 2). For SWCNT, the peak at 707.02 eV indicates the presence of Fe 2p, attributed to the process of obtaining SWCNT, given that transition metals from columns 8, 9, and 10 of the periodic table, such as iron, cobalt, and nickel, are used as catalysts in the growth of carbon nanotubes.^46^ The atomic percentage of iron present in the sample is 0.78%. No transition metals were detected in MWCNT. The percentage of oxygen impurities is slightly higher in SWCNT (4.01%) than in MWCNT (3.31%), as reported in Table 2.

**Figure 2:**
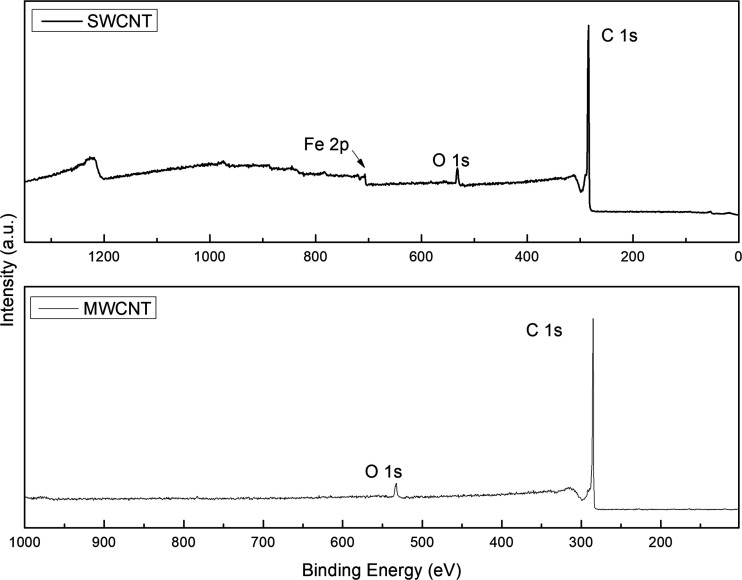
X-ray photoelectron spectroscopy (XPS) survey of SWCNT and MWCNT.

**Table 2:** Atomic Contribution and Binding Energy of Carbon, Oxygen, and Iron Obtained from the Survey Spectra of SWCNT and MWCNT

		% atomic		binding energy (eV)
SWCNT	C 1s	95.21	284.01	
	O 1s	4.01	532.01	
	Fe 2p	0.78	707.01	
MWCNT	C 1s	96.69	285.4	
	O 1s	3.31		532.9

Figure 3 shows the high-resolution spectra of carbon and oxygen for SWCNT and MWCNT. SWCNT presents characteristic peaks of C=C, C–C, and C–O bonds at 284.48, 284.63, and 285.84 eV, respectively. The shakeup π-π* peak at 289.82 eV is characteristic of sp^2^ carbons, a final state effect when an electron is excited to an unoccupied state by the energy transmission of a photoelectron.^47–49^ The oxygenated bonds of SWCNT were identified as COOH, C=O, and C–O, indicating the presence of acidic groups (−COOH) on the surface of the SWCNT. MWCNT shows the presence of COOH, C–OH, and C=O groups on its surface. Fewer acidic −COOH groups are present on the MWCNT surface when compared to the SWCNT (Table 3).

**Figure 3:**
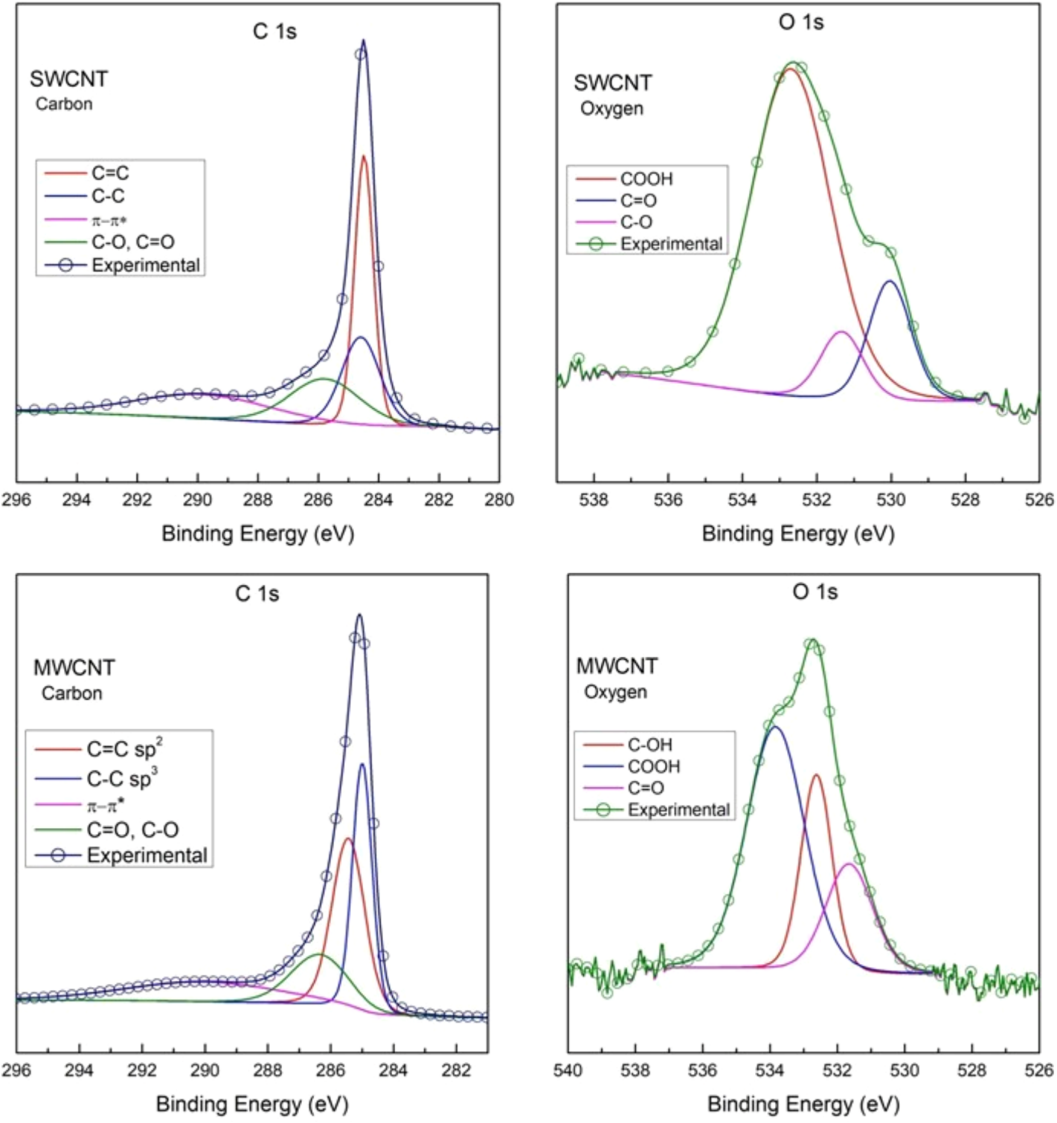
Deconvoluted XPS core level peaks of carbon 1s and oxygen 1s from SWCNT and MWCNT.

**Table 3:** Atomic Contribution and Binding Energy of Carbon and Oxygen Obtained from the Survey Spectra of SWCNT and MWCNT

		SWCNT		MWCNT	
		% atomic	binding energy (eV)	% atomic	binding energy (eV)
C 1s	C=C sp^2^	33.74	284.48	33.93	285.43
	C–C sp^3^	22.58	284.63	29.68	285.01
	C–O, C=O	21.86	285.84	17.57	286.35
	π-π*	21.82	289.82	18.82	290.03
O 1s	C=O	14.82	530.04	20.5	531.61
	C–O	8.61	531.33	24.19	532.6
	COOH	76.57	533.70	55.31	533.84

#### 3.1.3 Raman Spectroscopy

Raman spectra of SWCNT and MWCNT exhibit a characteristic band around 1593 and 1582 cm^–1^, respectively, known as the G band, which arises from the stretching vibrations of the sp^2^ hybridized lattice (Figure 4). The second characteristic band, centered at 1342 cm^–1^, is the D band, which represents defects in the sp^2^ hybridized systems. The third band, centered at 2662 and 2683 cm^–1^ for SWCNT and MWCNT, respectively, is the 2D band which is the first harmonic of the D band. The *I*_D_/*I*_G_ intensity ratio can be used as a qualitative tool to measure the level of disorder in the material. A ratio *I*_D_/*I*_G_ > 1 indicates a greater number of defects in the sp^2^ hybridized network, while *I*_D_/*I*_G_ < 1 indicates fewer defects in the material.^50,51^ According to Jorio and Saito the intensity of the D band is strongly influenced by the polarization of the laser wavelength used during the Raman analysis,^52^ suggesting that the observation of the D band in the Raman spectrum of MWCNTs may not always originate from the disorder present on the surface of the material. In a more recent work, Dresselhaus et al. showed that SWCNTs tend to exhibit low D band intensity, which can be explained by considering the one-dimensional character of the defects and the double resonance theory.^53^ The *I*_D_/*I*_G_ (dimensionless) values of SWCNT and MWCNT were 0.01 and 1.39, respectively. The SWCNT and MWCNT characteristics are summarized in Table 4.

**Figure 4:**
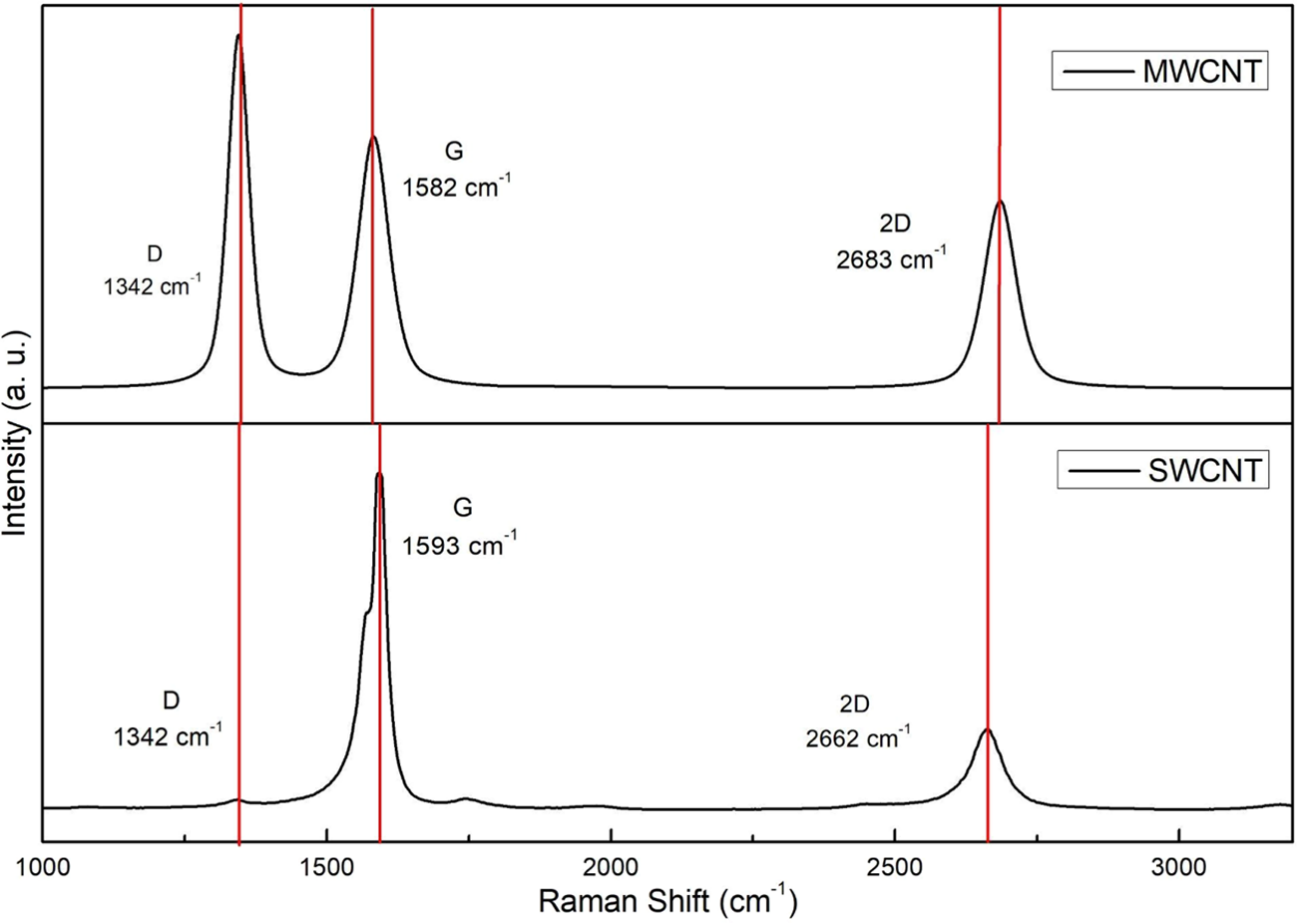
Raman spectra of SWCNT and MWCNT.

**Table 4:** Summary of the Characteristics of SWCNT and MWCNT

	SWCNT	MWCNT
external diameter (nm)	1.6 ± 0.4^a^	10–30^b^
length (μm)	5.0^a^	5–30^b^
surface area (mg·m^2^/g)	300^a^	>150^b^
purity (%)	87.1	>93
carbon content (%)	95.21	96.69
oxygen content (%)	4.01	3.31
metallic impurities (%)	Fe: 0.78	
carbon sp^2^ content (%)	33.74	33.93
*I*_D_/*I*_G_	0.01	1.39
electrical conductivity (S·cm^–1^)	4.66 × 10^–3^	1.12 × 10^–1^
price (US$/kg)	4000.00	860.00

a Data obtained from Tuball datasheet.

b Data obtained from Nanoview datasheet.

### 3.2 Characterization of Rubber Compounds

#### 3.2.1 Morphology and Microstructure

In rubber composites, silica and carbon nanotubes have a tendency to agglomerate due to their high surface energy when compared to nonpolar elastomers like NR and BR, making it difficult to incorporate, distribute, and disperse them in rubber matrices.^54^ In addition, the weak interaction between nanotubes and elastomers leads to ineffective dispersion and the formation of tangles.^55^

The SEM micrographs of silica-filled elastomer compounds containing SWCNT and MWCNT are shown in Figure 5.

**Figure 5:**
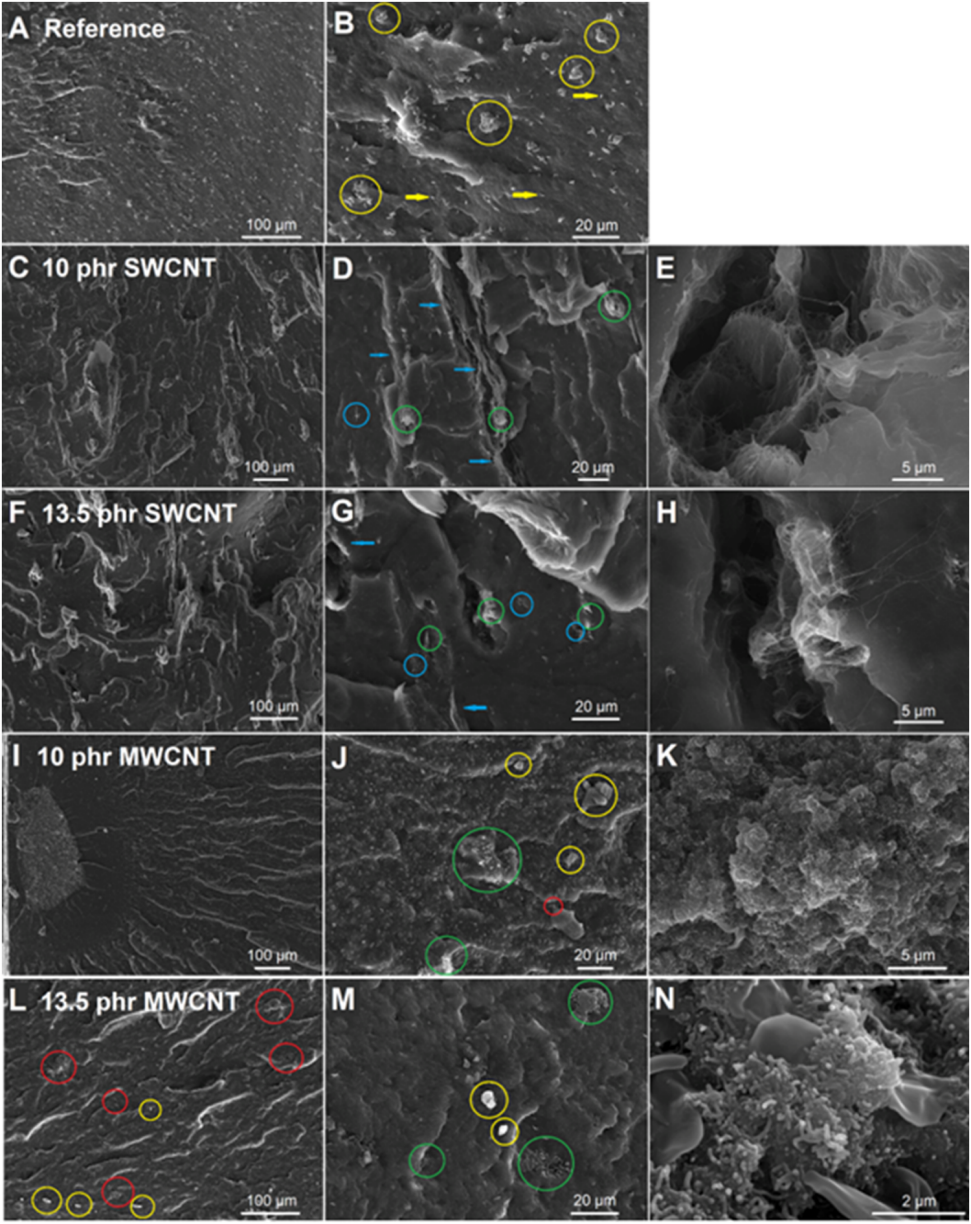
SEM micrographs of the sample without CNT (a and b), 10 phr SWCNT (c, d, and e), 13.5 phr SWCNT (f, g, and h), 10 phr MWCNT (i, j, and k), and 13.5 phr MWCNT (l, m, and n). (b) Apparent silica agglomerates are indicated by circles, and some isolated particles are pointed by yellow arrows. (d and g) Apparent SWCNT bundles are indicated by blue arrows, isolated SWCNTs by blue circles, and silica and SWCNT agglomerates by green circles. (e and h) Higher magnification of regions with SWCNT bundles (blue arrows). (j, l, and m) Apparent silica clusters are indicated by yellow circles, MWCNT agglomerates by red circles, and silica and MWCNT agglomerates by green circles. (k and n) Higher magnification of regions with silica and MWCNT agglomerates (green circles).

A good distribution of silica was observed in the reference sample without CNT (Figure 5a,5b). Isolated silica particles (yellow arrow) were observed, as well as silica agglomerates (circles). Figure 5c–5e show a 10 phr SWCNT sample. Elongated bundle structures were observed, indicated by blue arrows (Figure 5d). Grouped parallel SWCNT bundles create continuous threads, forming a microfibrillar structure in the rubber matrix, responsible for an interconnected network that is crucial to impart the nanocomposites with greater electrical conductivity.^56,57^ As the concentration of SWCNTs increases to 13.5 phr, there is a higher presence of voids and a lower presence of nanotube bundles (Figure 5g). Due to lower dispersion and distribution of nanotubes, there are agglomerates that prevent the formation of networks of entangled bundles, directly affecting the electrical conductivity of the sample.^58^

The initial morphology of MWCNTs differs greatly from SWCNTs (Figure 1), which affects the CNT network morphology in the compounds. For the 10 phr MWCNT sample, Figure 5i represents a region where an agglomerate of MWCNT is surrounded by an area with no visible MWCNT and silica particles, highlighting the poor dispersion and distribution of fillers. These regions with no MWCNTs tend to limit the material’s conductivity. In regions with a high concentration of MWCNTs and silica, the presence of agglomerates of silica (yellow circles), agglomerates of MWCNTs (red circles), and agglomerates of both silica and MWCNTs (green circles) was observed (Figure 5j). Increasing the content of MWCNT to 13.5 phr improves the distribution of both MWCNT and silica (Figure 5l–n). The few observed agglomerates (yellow circles) are smaller and better dispersed than in the reference sample (Figure 5a). Agglomerates of nanotubes can be seen in Figure 5m (green circles), and silica particles seem to be intimately associated with MWCNT agglomerates (Figure 5l).

Finally, there might be a heterogeneous distribution of CNTs between the two phases of the NR/BR blends that it is not possible to observe on the SEM images. To the best of our knowledge, no study has investigated the distribution of CNTs between phases in a NR/BR blend. Le et al. observed the distribution of MWCNTs in a ternary blend of styrene butadiene rubber (SBR)/nitrile butadiene rubber (NBR)/natural rubber (NR) and noticed a preferential localization of MWCNTs in the polar NBR and nonpolar NR, but not in the SBR phase.^59^ The preference of MWCNTs for the NR phase is due to the presence of phospholipids in NR, as the ammonium cation N^+^ of phospholipids interacts through cation-π interaction with the surface of MWCNTs. Thus, we can reasonably expect a heterogeneous distribution of SWCNTs and MWCNTs between the NR and BR phases, which may be responsible for the need of a relatively high amount of CNTs to achieve the desired electrical conductivity.

#### 3.2.2 Electrical Conductivity

Carbon nanotubes have great potential to promote electrical conductivity in polymeric materials, including elastomers, due to their high aspect ratio.^57^ Figure 6 shows the behavior of the AC conductivity of samples as a function of the concentration of SWCNT and MWCNT. The percolation threshold for SWCNT appears to fall within the 5 to 10 phr nanotube concentration, demonstrating a substantial increase in conductivity compared to the reference sample that contains only silica. Notably, the most significant jump in conductivity occurs from 8 to 10 phr, reaching its highest conductivity for SWCNT samples at 10 phr (1.5 × 10^–5^ S/m). The 13.5 phr SWCNT sample exhibits a decline in conductivity, which can be explained by the poor nanotube dispersion at this concentration or reagglomeration and presence of voids (Figure 5f–h). Conversely, samples containing MWCNT exhibit the typical S-shaped percolation curve. The percolation threshold for MWCNT falls within a similar concentration range as the SWCNT, between 4 and 13.5 phr, with the most significant conductivity jump observed from 10 to 13.5 phr. These results indicate that percolation occurs at slightly lower concentrations for SWCNT than for MWCNT samples. This difference in percolation concentration can be attributed to the different morphology of the two kinds of nanotubes, as observed in Figure 5. Conductivity surges when the long microfibrils of SWCNT form an interconnected network within the matrix, as observed on the SEM images of the 10 phr SWCNT (Figure 5c–e). In the case of MWCNTs, they form entanglements composed of curved individual nanotubes, and that geometry makes it more difficult to establish a continuous conductive pathway.^56^

**Figure 6:**
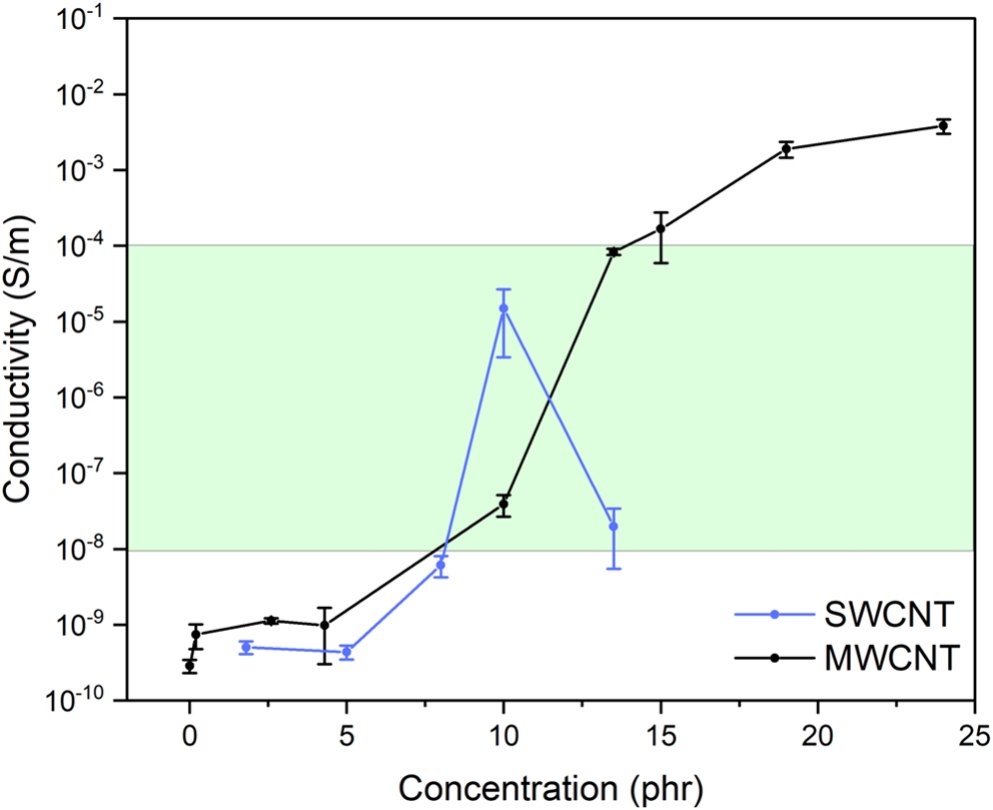
Percolation curves of SWCNT and MWCNT. The green region represents the range of antistatic conductivity as established by ASTM D991.

Following the ASTM D991 standard, antistatic rubber products are expected to demonstrate specific electrical conductivity falling within the range of 10^–4^ to 10^–8^ S·m^–1^.^11,19^ Thus, SWCNT and MWCNT exhibit antistatic properties adequate for tire thread application at 10 and 13.5 phr, corresponding to 5.3 to 7 wt %, respectively.

Despite achieving the desired conductivity range with a relatively high concentration of nanoparticles, these results are consistent with those reported in the literature, which generally indicates that up to 10 wt % of CNTs are necessary to achieve a conductivity threshold.^60–62^ Different factors can explain the necessity for high concentration of CNTs, such as the difficulty of CNT dispersion and distribution in the NR/BR matrix using the internal mixer technique, as observed on SEM images, the *I*_D_/*I*_G_ ratio, and the diameter of the nanoparticles. In their work, Gan et al. observed that the best results were obtained for MWCNT with an *I*_D_/*I*_G_ ratio of 1.26 and an average diameter of 9.5 nm. The excessive presence of defects and larger diameters hinders the formation of a percolation network, increasing the concentration required to reach the percolation threshold.^63^ In comparison, the *I*_D_/*I*_G_ of MWCNTs used in this work is 1.39, and the external diameter is between 10 and 30 nm. Thus, the high level of defects and larger diameter, combined with the high entanglement of the MWCNTs, may have influenced the critical concentration required for percolation. Moreover, the high silica loading might have affected the conductivity by disturbing/interfering with the CNT network and interrupting the conductive pathway since silica is an insulator.^33^ Therefore, nanocomposites exhibiting desired electrical conductivities contain a high concentration of nanofillers (10 phr for SWCNT and 13.5 phr for MWCNT), and a high precipitated silica content (55 phr) as well.

#### 3.2.3 Vulcanization Kinetics

The vulcanization curves of the nanocomposites cured at 170 °C are presented in Figure 7, and the scorch time (*t*_s_), optimum cure time (*t*_90_), maximum torque (*M*_H_), and minimum torque (*M*_L_) are reported in Table 5.

**Figure 7:**
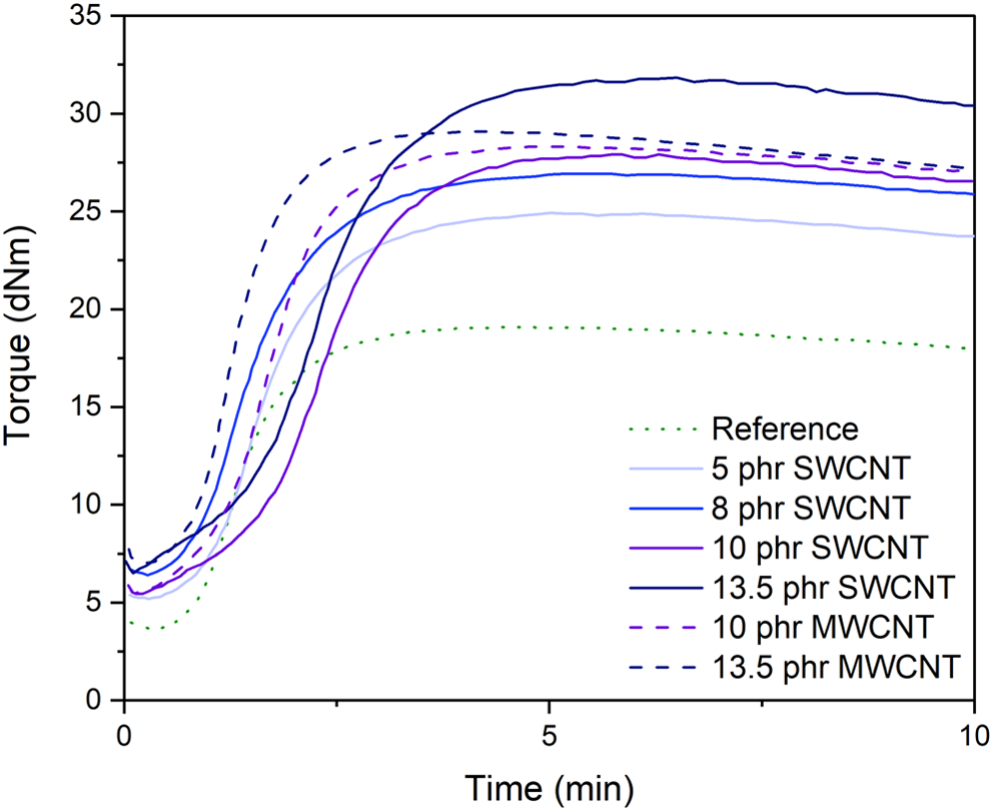
Rheometric curves of NR/BR compounds reinforced with silica (55 phr) and different contents of SWCNT and MWCNT.

**Table 5:** Cure Properties of the NR/BR Compounds Reinforced with Silica and Different Contents of SWCNT and MWCNT

sample	*M*_L_ (dNm)	*M*_H_ (dNm)	*M*_L_–*M*_H_ (dNm)	*t*_s_ (min:s)	*t*_90_ (min:s)
reference	3.65	19.10	15.45	00:56	02:22
5 phr SWCNT	5.21	24.90	19.69	00:59	02:52
8 phr SWCNT	6.45	26.95	20.50	00:50	02:49
10 phr SWCNT	5.44	27.88	22.44	01:02	03:30
13.5 phr SWCNT	6.44	31.74	25.30	00:47	03:36
10 phr MWCNT	5.50	28.37	22.87	00:50	02:42
13.5 phr MWCNT	7.12	29.08	21.96	00:47	02:10

For the reference sample containing only silica, *t*_s_ is 56 s, and *t*_90_ is 2:22 min. Notably, the addition of up to 10 phr of SWCNTs results in low variations of *t*_s_ but in an increase of *t*_90_, whereas for 13.5 phr, *t*_s_ decreases and *t*_90_ remains similar. According to the literature, conflicting effects of nanotubes on vulcanization are observed. The hollow structure of nanotubes is known to absorb curing agents, and certain accelerators that contain aromatic rings, such as TBBS, may be adsorbed on the nanotube surface, inhibiting their dissociation and delaying the curing process.^64^ Furthermore, acidic groups on the surface of SWCNTs (Table 3) can interact with radicals generated during vulcanization, impacting the process.^64^ On the other hand, metallic impurities on SWCNT, such as Fe, may catalyze vulcanization.^65,66^ Enhanced thermal conductivity due to nanotube dispersion and percolation can also contribute to reducing *t*_s_ and *t*_90_.^31,67^ Additionally, both nanotubes and silica can increase the compound’s viscosity during the final mixing stage, when the vulcanization agents are incorporated. This increase in viscosity can hinder the dispersion of these additives and consequently slow down the vulcanization process.^68^

The presence of MWCNT at 10 and 13.5 phr decreases *t*_s_ in comparison to the reference sample. When comparing the 10 phr SWCNT and 13.5 phr MWCNT samples, both exhibit high conductivity, ∼10^–5^ and 10^–4^ S·m^–1^, respectively, and a well-dispersed morphology in the elastomer. However, MWCNT shows a lower scorch time and faster curing kinetics. Given that thermal conductivity is expected to follow a similar trend as the electrical conductivity for both samples, and that the viscosity of 13.5 phr MWCNT is higher than that of 10 phr SWCNT in the final mixing stage (evolution of the torque of the mixer rotor is presented in Supporting Information A and B), the faster vulcanization kinetics of 13.5 phr MWCNT may be accounted by the lower surface area and fewer COOH and OH groups, which may limit accelerator adsorption.

The incorporation of SWCNTs notably increases both *M*_L_ and *M*_H_ values, likely due to the nanotube network formation within the compound, which increases the viscosity.^60^ The increase in *M*_H_ can be attributed to the reinforcing effect of SWCNTs, which results in a higher cross-link density that restricts molecular mobility in the polymer matrix.^30,69,70^ As observed for SWCNT, *M*_L_ and *M*_H_ increase with increasing MWCNT content. However, the *M*_L_–*M*_H_ values are similar for 10 and 13.5 phr MWCNT. This may be attributed to the increase in viscosity caused by the higher MWCNT content, even though the silica and MWCNT dispersion is improved, as observed in the SEM images.

Increases in viscosity of the compounds due to nanofillers can also be observed in the torque values during the second and last mixing stages, which are higher than for the reference sample (see Supporting Information A, B, and C). After the incorporation of CNTs, torque rose, as well as the temperature of the compound.

#### 3.2.4 Payne Effect

The Payne effect was analyzed based on the amplitude of the decrease in the storage modulus between 0 and 28% of deformation (Figure 8). At lower deformations, it provides insights into filler–rubber interactions, while the filler–filler network is assessed at higher deformations.^5,71,72^

**Figure 8:**
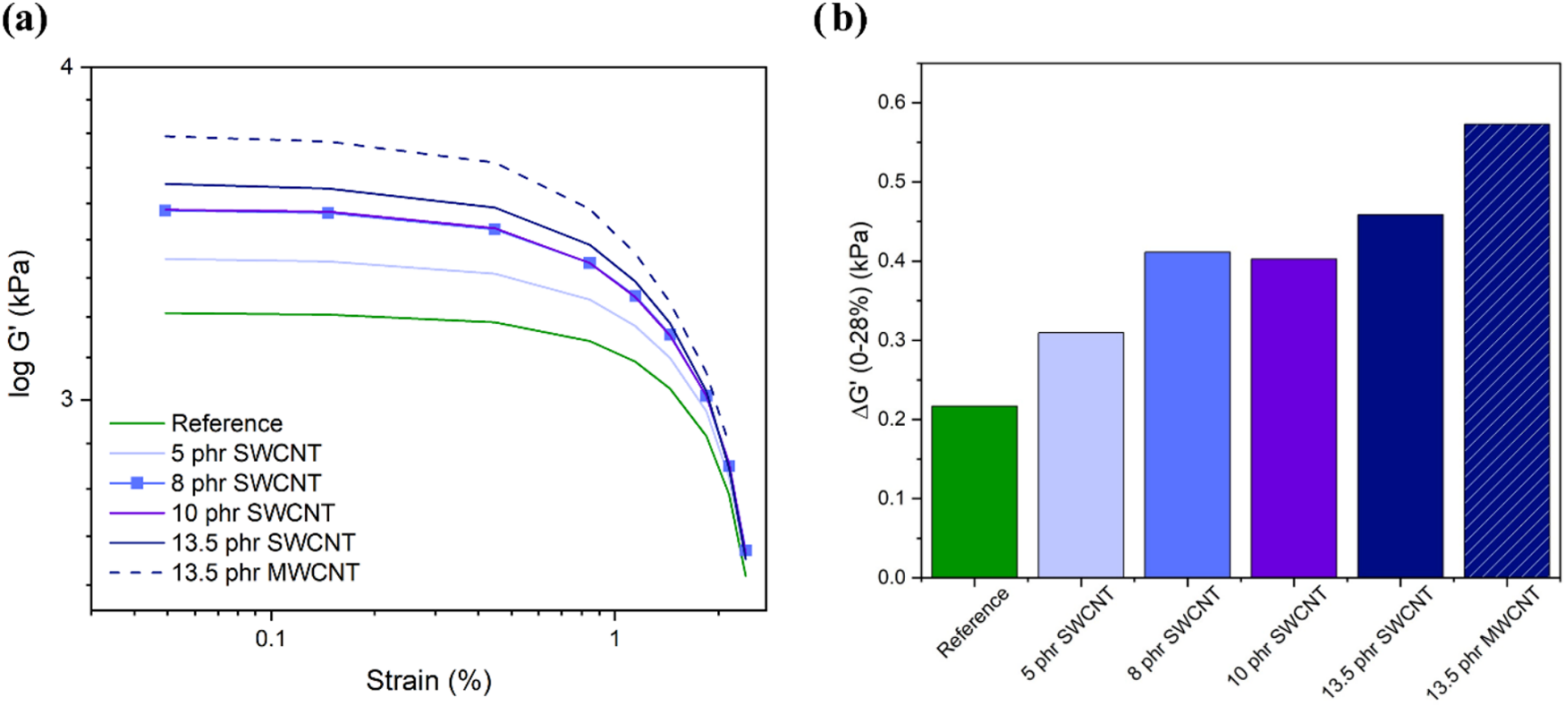
Payne effect: (a) Evolution of the storage modulus *G*′ with strain and (b) Evolution of Δ*G*′.

The reference sample presents the Payne effect due to the rupture of the silica filler network when subjected to high deformations, which leads to the release of the elastomer fraction trapped in the filler aggregates. Silica has a well-known strong tendency to aggregate,^16^ and silanization has not avoided this phenomenon. In the samples with CNTs, the Payne effect is amplified with increasing nanotube content. The drop of *G*′ is due to the breakdown of the nanotube network, the breakdown of CNT aggregates, the release of the trapped elastomer in CNT aggregates, and the filler/rubber bonding and debonding.^29,31,73,74^

The 10 phr SWCNT sample exhibits a Payne effect similar to the 8 phr SWCNT sample, which evidence the good dispersion at 10 phr. The Payne effect for 13.5 phr SWCNT is more prominent due to poorer nanotube dispersion and aggregate breakdown during the test. Regarding the types of nanotubes, samples with similar MWCNT concentrations exhibit a higher Payne effect than those with SWCNT. This difference in the Payne effect may be attributed to the morphology of the aggregates: while SWCNTs tend to align in a preferential direction, MWCNTs form entangled structures (Figure 1), leading to more intense network breakage.

#### 3.2.5 Dynamic mechanical analysis

DMA was performed by subjecting the samples to a temperature sweep in tensile loading mode from −80 to 100 °C at a frequency of 10 Hz. The evolution of the storage modulus and loss factor tan δ as function of temperature is reported in Figure 9 for all the samples. The glass transition of polybutadiene was not observed in the temperature range tested since it is reported to occur around −90 °C.^41,75,76^

**Figure 9:**
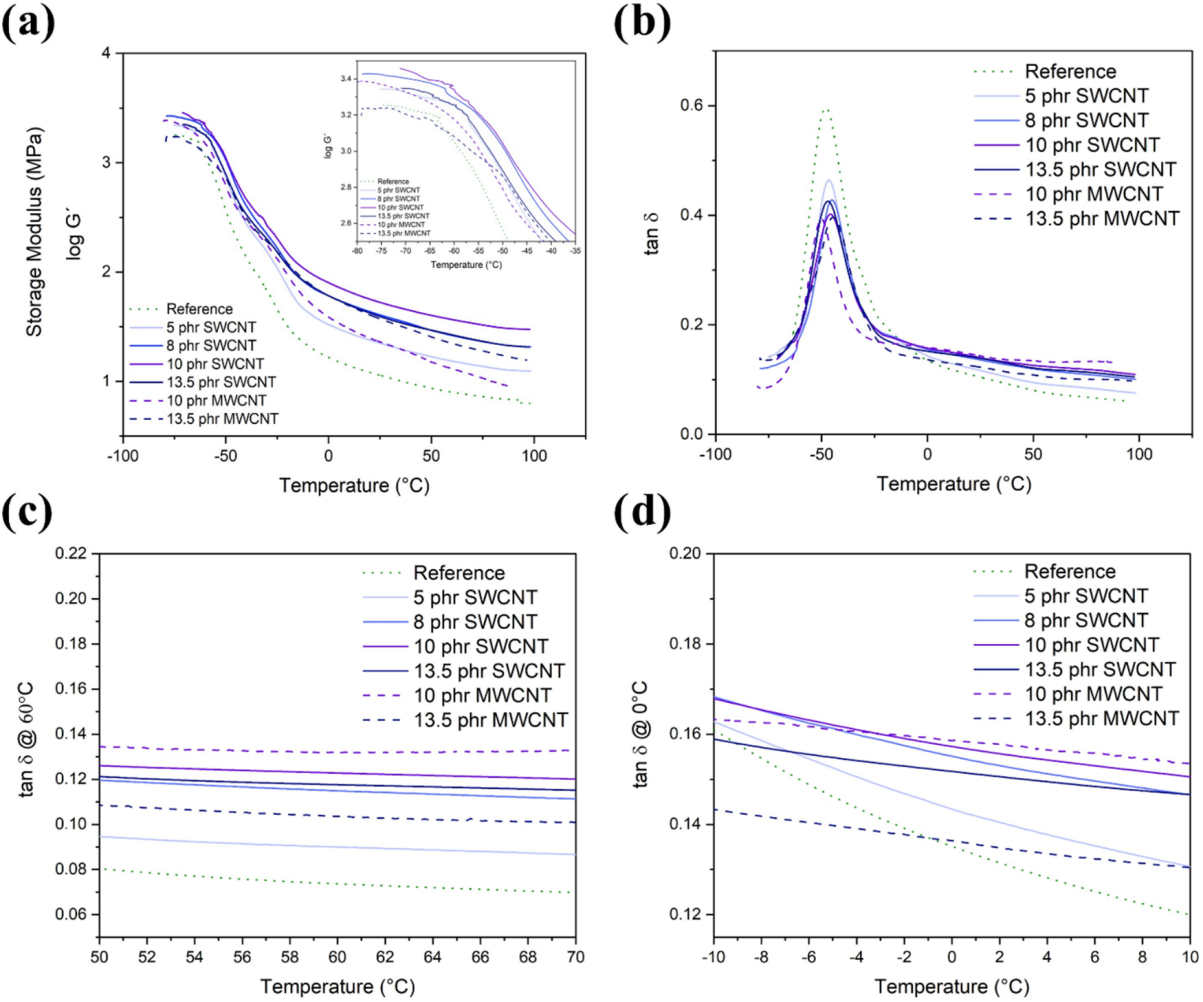
Dynamic-mechanical analysis: (a) storage modulus, (b) loss factor (tan δ) as a function of temperature; (c) value of loss factor at 60 °C to evaluate the rolling resistance; and (d) value of loss factor at 0 °C to evaluate the wet grip, as a function of temperature.

The storage modulus (*G*′) is high at low temperatures, corresponding to the rubber in its glassy state. When reaching the glass–rubber transition, it suddenly drops due to an energy dissipation mechanism (Figure 9a). During this transition, the loss factor exhibits a peak, related to the glass transition temperature (*T*_g_) of the natural rubber (Figure 9b).

The low-temperature *G*′ increases drastically upon the addition of SWCNT, whereas the change is less prominent for MWCNT: while the sample with 10 phr presented a slight increase, the sample with 13.5 phr showed almost the same modulus as the reference. The increase in the content of SWCNT up to 10 phr also promotes an increase in *G*′, whereas a decrease in *G*′ is observed for 13.5 phr, becoming similar to the 5 phr *G*′ values. The increase of *G*′ in the glassy plateau region is related to a lower mobility of the rubber chains that are immobilized on the large-surface area of CNT,^77–79^ which causes an efficient charge transfer between the rubber and the rigid nanofiller. This is enhanced by increasing the CNT content and improving its dispersion. On the contrary, the drop observed at 13.5 phr is correlated to the aggregation of SWCNT and the hydrodynamic amplification effect,^29,77^ which is consistent with the results observed by SEM (Figure 5).

In the rubbery state, the nanocomposites’ storage modulus with different SWCNT content follows the same trend. Compared to the samples with 10 and 13.5 phr of MWCNT, the 10 phr SWCNT sample exhibits the highest *G*′ values. Therefore, it can be concluded that SWCNT has a higher reinforcing effect for NR/BR than MWCNT due to their intrinsic behavior and also higher surface area (Table 4).^46^

Figure 9b represents the loss factor as a function of temperature for all the samples. The peak of tan δ corresponds to the energy dissipation at *T*_g_ of the rubber and is centered at −46.3 °C for the reference sample. The *T*_g_ of nanocomposites does not significantly differ from the reference compound (Table 6), which is coherent with Bakošová and Bakošová work.^28^ Although one sample (10 phr MWCNT) showed a reduction in the *T*_g_ peak, no significant change in *T*_g_ was observed in any other sample, which leads us to believe that this is a specific behavior of this sample and does not translate into a trend toward the use of CNT in NR/BR elastomeric matrices. However, the clear tan δ peak height reduction is attributed to the reduced mobility of rubber due to CNT presence, which is related to the volume of rubber trapped in the filler aggregates (silica and CNT), and the increased volume of rubber immobilized on the surface of CNT.^60^ The decrease in the width at half height of the tan δ peak as the CNT content increases also corroborates the higher polymer fraction bound tightly to the CNT surface explanation. The higher reinforcing effect of SWCNT compared to MWCNT can also be confirmed since the tan δ peak of the 10 phr SWCNT sample almost overlaps with that of 13.5 phr MWCNT.

**Table 6:** Glass Transition Values and Tan δ Values at 0 and 60 °C Obtained by DMA

sample	*T*_g_ (°C)	tan δ @ 0 °C	tan δ @ 60 °C
reference	–46.3 ± 2.5	0.1431 ± 0.0124	0.0757 ± 0.0086
5 phr SWCNT	–46.6 ± 0.3	0.1424 ± 0.0040	0.0936 ± 0.0058
8 phr SWCNT	–45.4 ± 0.4	0.1556 ± 0.0005	0.113 ± 0.0020
10 phr SWCNT	–45.4 ± 0.7	0.1559 ± 0.0026	0.1208 ± 0.0018
13.5 phr SWCNT	–46.3 ± 0.9	0.1519 ± 0.0003	0.1187 ± 0.0010
10 phr MWCNT	–50.3 ± 0.5	0.1629 ± 0.0049	0.1368 ± 0.0086
13.5 phr MWCNT	–45.4 ± 0.6	0.1358 ± 0.0081	0.1024 ± 0.0016

##### 3.2.5.1 Rolling Resistance

The performance of elastomeric compounds for tire tread can be estimated by DMA. The relative values of tan δ at 60 °C (tan δ@60 °C) at low deformation and high frequencies (between 10 to 100 Hz) permit the evaluation of the rolling resistance since it simulates the conditions experienced by a tire tread during use. A decrease of the tan δ@60 °C is associated with lower rolling resistance and, consequently, a decrease in fuel consumption.^80^ Compared to the reference sample, the nanocomposites exhibited a higher tan δ, which tended to increase with the content of nanotubes.

For SWCNT, the increase in nanotube content resulted in a consistent increase in rolling resistance up to 10 phr, whereas the tan δ@60 °C values were similar at 10 and 13.5 phr and 59.6 and 56.8% higher than the compound without CNT, respectively. Comparing SWCNT and MWCNT at 13.5 phr, lower rolling resistance was obtained for MWCNT, which can be attributed to a better nanotube dispersion, since the sample 13.5 phr SWCNT showed poor dispersion. Besides, if comparing only the samples with good dispersion state, 10 phr SWCNT and 13.5 phr MWCNT, the MWCNT sample shows lower rolling resistance, which confirms that MWCNT is a good candidate for fuel economy.

The increase in the rolling resistance is correlated to an increase in energy dissipation and has been reported elsewhere for rubber nanocomposites containing CNT.^60^ Different mechanisms are responsible for this energy dissipation, such as the rubber bonding-debonding occurring under dynamic deformation, friction caused by filler–rubber–filler due to the presence of filler aggregates and agglomerates that may not be disrupted during dynamic deformation, and dissipative phenomena related to the rubber network (that is affected by the presence of high CNT content that decreases the cross-linking density, and consequently, the elastic behavior of this portion of the rubber). Combining ^1^H DQ-NMR and equilibrium swelling experiments, Bernal-Ortega et al.^60^ concluded that the fraction of rubber interacting with the MWCNT surface is more than 5-times higher than the effect of carbon black and thus it highly contributes to the rubber bonding-debonding on the CNT surface.^50^ Even so, the stronger interaction between MWCNT and the polymer molecules, due to the lower presence of polar groups at the surface, may be the cause for better results when compared with SWCNT.

##### 3.2.5.2 Wet Grip

The wet grip is an important property for tire tread since it is related to drivability and safety. The wet grip can be evaluated by tan δ values at 0 °C (tan δ@0 °C), where a high value corresponds to a high wet grip, and also related with the peak height, as the immobility of polymer molecules acts as a barrier to the deformation of the tread tires, resulting in lower grip.^81^ Compared with the reference sample, the addition of both types of CNT improves not only the immobility of polymer molecules as discussed earlier, but also values of tan δ@0 °C. The addition of SWCNT improved values of tan δ@0 °C of Si-filled NR/BR, particularly when increasing SWCNT content and its dispersion (improvement by 8.9% at 10 phr SWCNT), whereas the sample with 13.5 phr MWCNT exhibited a tan δ@0 °C similar to the reference sample. Therefore, by promoting a higher tan δ@0 °C and higher peak height, SWCNTs seems to be a better choice for wet grip performance than MWCNT.

#### 3.2.6 Tensile Properties

Stress–strain tensile curves are reported in Figure 10. The ultimate tensile strength (UTS), modulus at 50% (M50%), 100% (M100%), and 300% (M300%), and the elongation at break are reported in Table 7. The elastic moduli can be related to the material’s stiffness, and the ratio between M300% and M100%, known as the reinforcement index, is usually used to understand the filler’s influence on the stress–strain behavior of elastomers.

**Figure 10:**
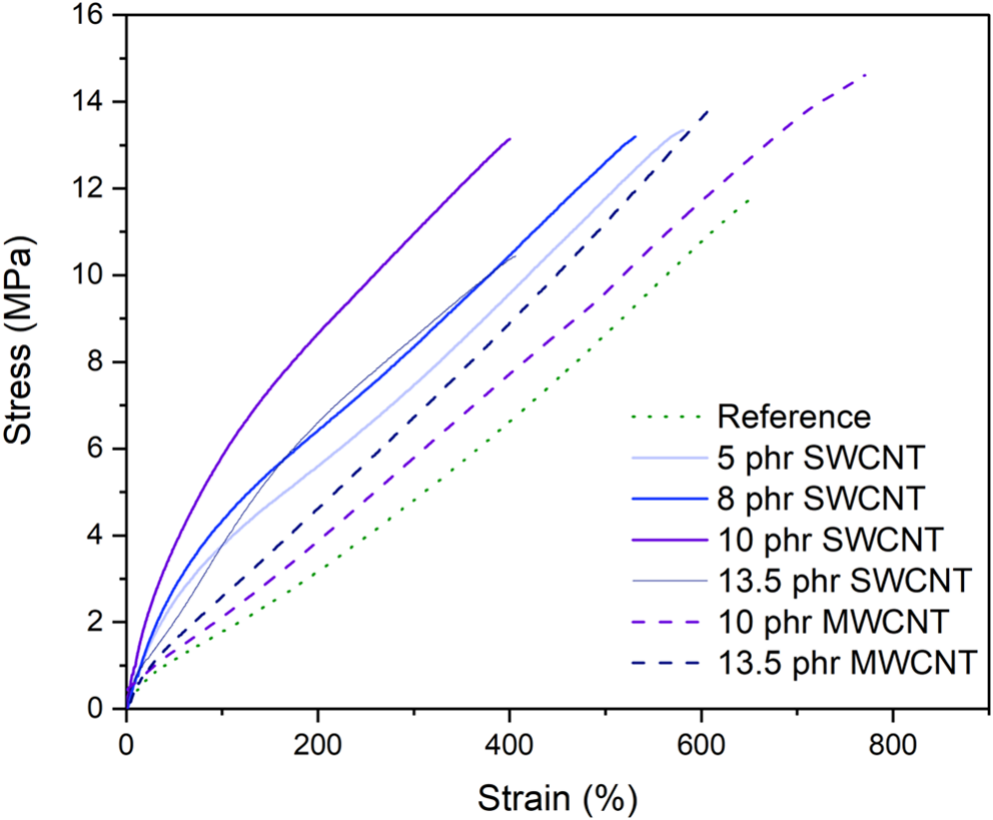
Stress–strain curves of the nanocomposites.

**Table 7:** Data Extracted from Stress-Strain Curves

sample	modulus at 50% (M50%) (MPa)	modulus at 100% (M100%) (MPa)	modulus at 300% (M300%) (MPa)	reinforcement index (M300%/M100%)	ultimate tensile strength (UTS) (MPa)	elongation at break (%)
reference	1.1 ± 0.01	1.6 ± 0.2	4.4 ± 0.6	2.83	14.3 ± 0.5	858.0 ± 5.6
5 phr SWCNT	2.5 ± 0.01	3.8 ± 0.02	7.4 ± 0.1	2.01	7.3 ± 1.4	562.0 ± 66.7
8 phr SWCNT	2.8 ± 0.3	4.4 ± 0.1	8.0 ± 0.3	1.94	10.7 ± 2.5	551.0 ± 45.5
10 phr SWCNT	3.5 ± 0.1	5.8 ± 0.7	10.9 ± 0.7	1.88	13.1 ± 0.8	412.1 ± 11.2
13.5 phr SWCNT	2.2 ± 0.3	4.2 ± 0.7	9.1 ± 0.8	2.32	10.8 ± 1.1	407.3 ± 7.5
10 phr MWCNT	1.4 ± 0.1	2.2 ± 0.1	5.8 ± 0.1	2.62	14.9 ± 0.2	830.8 ± 71.9
13.5 phr MWCNT	1.5 ± 0.01	2.5 ± 0.03	6.6 ± 0.1	2.68	13.8 ± 0.1	609.0 ± 2.1

In general, the addition of CNT leads to an increase in elastic modulus and a reduction in elongation at break. This behavior is attributed to the reduced mobility of polymer molecules trapped on the filler surface by van der Waals interactions. The stress is transferred from the matrix to the rigid particles, restricting the flow and movement of polymer molecules, resulting in lower elongation at break.^82^ The larger the specific area of the filler, the greater the number of contacts that form between the filler and the polymeric matrix, thereby promoting the reduction in elongation at break.^83,84^

Considering the samples with SWCNT and antistatic conductivity, the sample with the highest SWCNT content (13.5 phr SWCNT) exhibited lower elastic moduli compared to the sample with 10 phr SWCNT. This trend is consistent with other results, such as the storage modulus obtained by DMA. At higher concentrations, SWCNT tends to aggregate, leading to a heterogeneous dispersion, which in turn results in a decrease of the mechanical properties. Furthermore, high filler concentrations can increase viscosity, resulting in voids and defects within the polymeric matrix.^85^

From a technological point of view, the addition of SWCNT decreased the reinforcement index (M300%/M100%). On one hand, this parameter assumes that the elastic modulus at 100% elongation (M100%) reflects the material′s stiffness as a result of its percolated filler network. On the other hand, the modulus at higher deformation (M300%) considers the rupture of the previously percolated network, assuming that the elastic modulus (M300%) is primarily influenced by the stiffness of the matrix. Indeed, SWCNT incorporation effectively increased the modulus at lower deformations, as observed in Table 7.^86^ However, for MWCNT-containing composites, the samples showed lower stiffness than those with SWCNT, but a higher reinforcement index. In general, both CNT types showed the same trend, with an increase in the elastic moduli proportional to the CNT concentration, while elongation at break and UTS were reduced for higher filler loadings.

Comparing composite pairs with the same CNT concentration (10 and 13.5 phr), SWCNT-containing samples exhibited higher elastic moduli. At 10 phr, SWCNT increases the modulus at M100% to 5.8 MPa (263% over the reference (1.6 MPa)), surpassing MWCNT’s M100% of 2.2 MPa (38% gain) at the same loading. This behavior might be a consequence of the higher anisotropic effect and strength, typical of SWCNT, corroborating the results that show a higher reinforcement effect for this type of CNT. SWCNTs typically form straight, fiber-like bundles that effectively reinforce composite materials, especially at lower strains, and exhibit a higher surface area, which enhances filler–polymer interactions and immobilizes more rubber chains, in accordance with a reduced tan δ peak height observed in Figure 9.b. However, their alignment reduces elongation at break and, consequently, the UTS. In contrast, the curly, entangled structure of MWCNTs is less effective in reinforcing the material at lower strains, becoming more effective at higher strains, leading to higher elongation at break, ultimate strength, and reinforcement index. Additionally, MWCNTs may exhibit greater affinity with the polymeric matrix, due to their lower surface concentration of polar groups, resulting in stronger filler–polymer interaction and more effective stress transfer. Furthermore, MWCNT leads to higher strain-induced crystallization of natural rubber, contributing to higher stress at break.^87^

#### 3.2.7 Hardness

The Shore D hardness values are shown in Figure 11. All samples containing CNT presented an increase in hardness when compared to the reference sample. The hardness was proportional to the CNT concentration, due to higher filler–filler and filler–polymer interactions, increasing the restriction on the movement of macromolecules.^88,89^ For the SWCNT samples, hardness values remain similar between the 10 and 13.5 phr CNT compositions. This is consistent with the poor dispersion of SWCNT at 13.5 phr observed previously. The samples with better dispersion state, 10 phr SWCNT and 13.5 phr MWCNT, showed an increase of 28% and 20% in Shore D hardness, respectively. The maximum hardness increase is obtained for 10 phr MWCNT, reaching 42% compared to the reference sample, which is coherent with data reported in the literature.^37,56^

**Figure 11:**
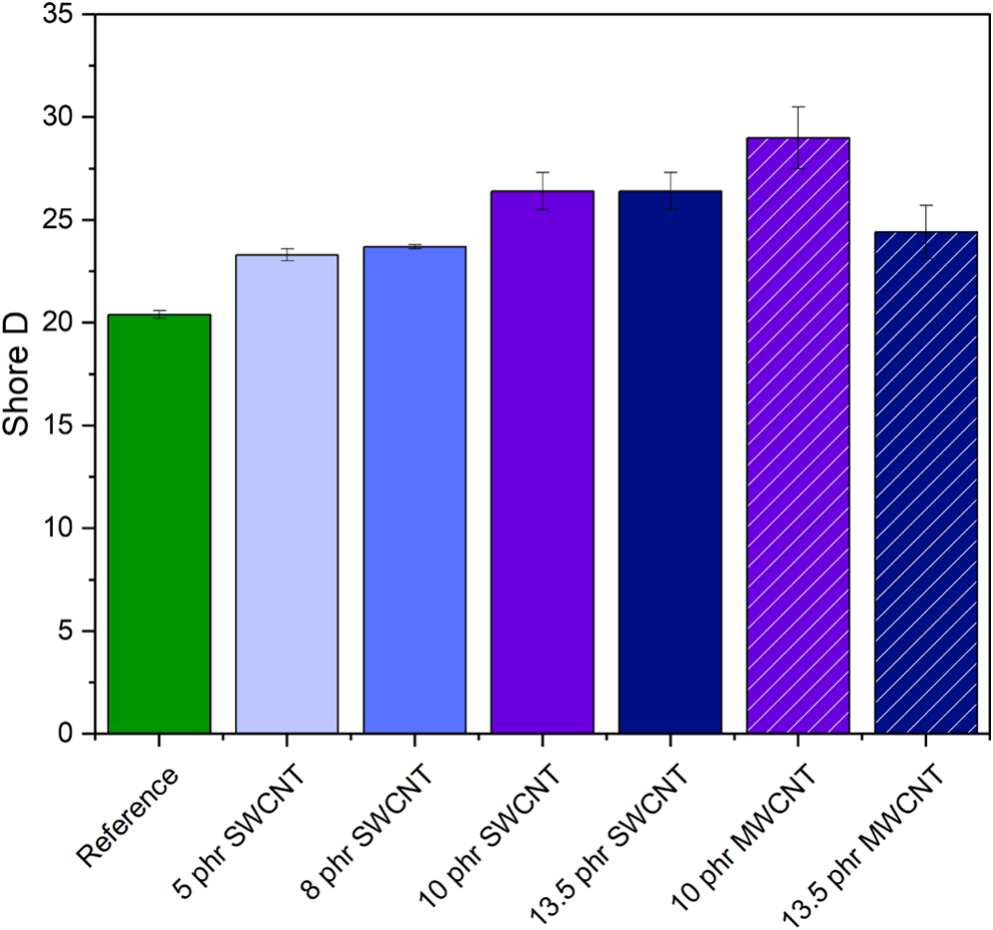
Shore D hardness.

## 4 Conclusions

This study explored pathways to enhance the performance of conductive green tire treads through the incorporation of single-walled carbon nanotubes (SWCNT) and multiwalled carbon nanotubes (MWCNT) into natural rubber/polybutadiene (NR/BR 1:1) composites reinforced with 55 phr of silica. The investigation revealed that the distinct morphological and compositional characteristics of SWCNT and MWCNT significantly influenced the composite properties, with the dispersion state of nanofillers being a pivotal factor on composite performance for both types of CNT. SWCNT exhibited an interconnected microfibrillar network, achieving antistatic-level conductivity at 10 phr, while MWCNT exhibited entangled individual coiled nanotubes, requiring higher loadings (13.5 phr) to reach the percolation threshold for electrical conductivity. SWCNT provided superior reinforcement, reducing polymer mobility more effectively, whereas MWCNT supported higher elongation and ultimate strength. For tire applications, both CNTs improved rolling resistance, with MWCNT-filled samples with 13.5 phr showing the best performance among the conductive samples. Despite SWCNT’s advantages in conductivity and reinforcement, its dispersion challenges and higher cost are still disadvantages when compared to MWCNT’s practical dispersion and cost-effectiveness. This study offered insights into optimizing conductive elastomer composites for energy-efficient tire technologies, filling a gap in the literature on the performance of CNT-filled green tires tread reinforced with high silica loadings prepared in the melt state using traditional mixing equipment.

## Supplementary Material



## Notes

Additional information includes processing curves (evolution of torque during the second and final mixing stage) of three different samples: 10 phr SWCNT (Supporting Information A), 13.5 phr MWCNT (Supporting Information B), and reference (Supporting Information C) (PDF)
