# Supplementary material for: Single-Walled and Multiwalled Carbon Nanotubes in Polybutadiene/Natural Rubber Composites Containing Silica for Conductive Green-Tire Application
Source: ACS Omega. 2025 Jul 1;10(27):29241–55. doi: 10.1021/acsomega.5c01638 (PMC12268747; doi:10.1021/acsomega.5c01638)
Supplement: Supplementary file 1 [file ao5c01638_si_001.pdf]

Supporting Information

Single-walled and Multi-walled Carbon Nanotubes in Polybutadiene/Natural Rubber Composites Containing Silica for Conductive Green Tire Application

Priscila Almeida Lucio Campini [a], Felipe Gustavo Ornaghi [a], Marcus Vinicius Braum [b], Elyff Cargnin [a], Guilherme Barnez Gramcianinov [a], Diego Moreira Lima [a], Renata dos Santos Pereira [b], Demétrio Jackson dos Santos [a], Anne Cristine Chinellato [a], Suel Eric Vidotti [a], Danilo Justino Carastan [a], Mathilde Champeau [a]*

[a] *Center of Engineering, Modeling and Applied Social Sciences, Federal University of ABC, Santo André, SP 09210-580, Brazil.*

[b] *PROMETEON TYRE GROUP, Av. Alexandre de Gusmão, 487 - Vila Homero Thon, Santo André - SP, 09111-310, Brazil*

* Corresponding author: mathilde.champeau@ufabc.edu.br

Supplementary information A. *Torque and temperature evolution during the second and last stages of mixture for 10 phr SWCNT*

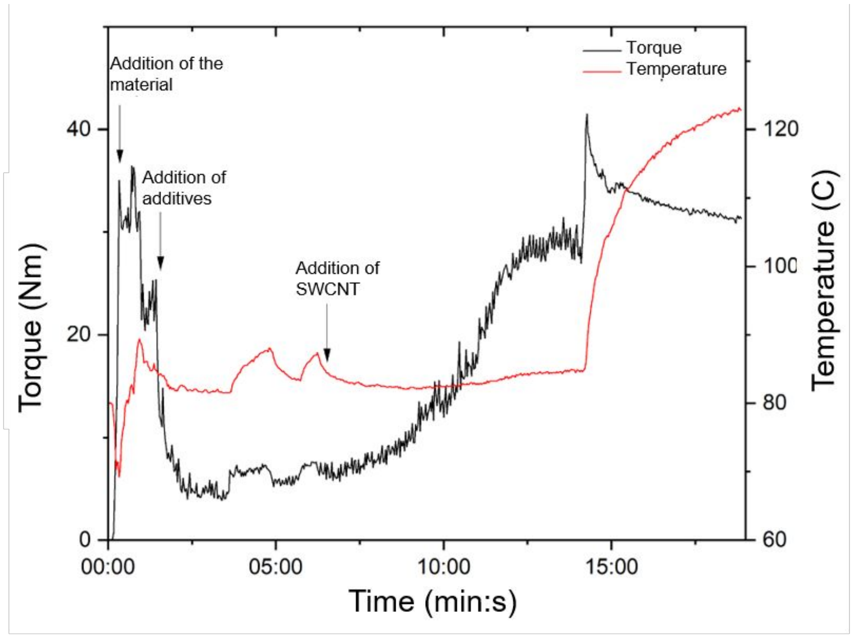

Figure S1. Torque and temperature evolution during the second stage mixture for 10 phr SWCNT

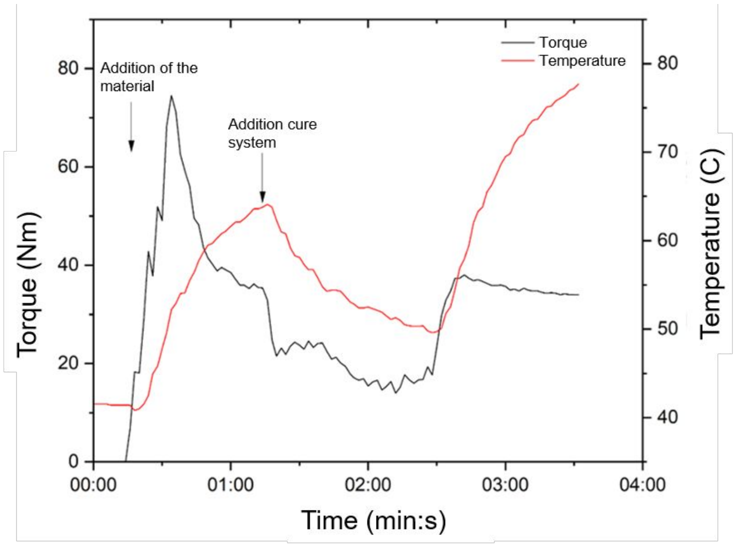

Figure S2: Torque and temperature evolution during the last stage mixture for 10 phr SWCNT.

Supplementary information B. *Torque and temperature evolution during the second and last stages of mixture for 13.5 phr MWCNT*

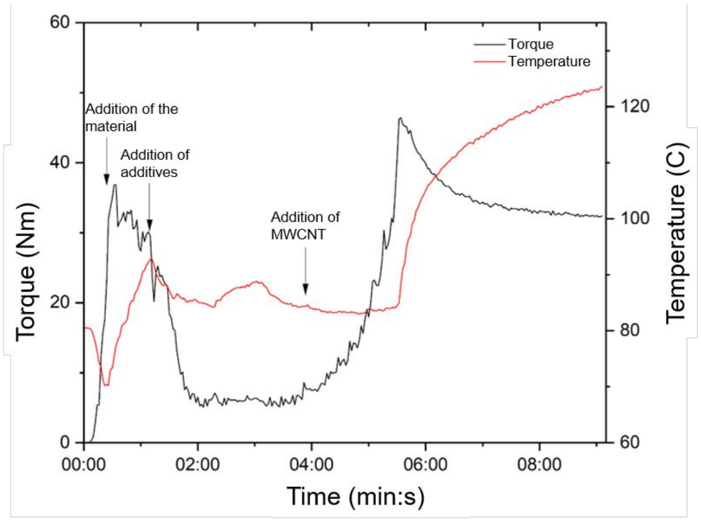

Figure S3. Torque and temperature evolution during the second stage mixture for 13.5 phr MWCNT

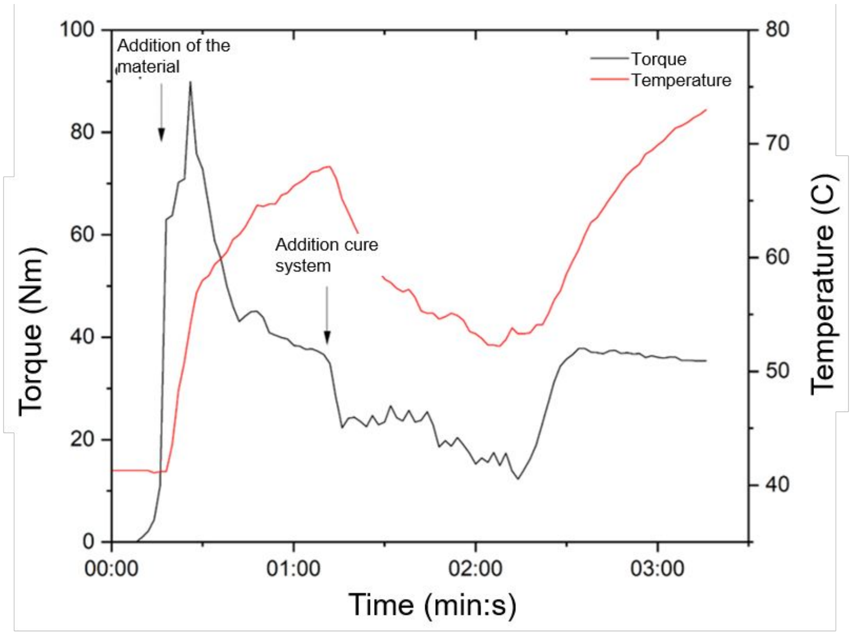

Figure S4. Torque and temperature evolution during the last stage mixture for 13.5 phr MWCNT.

Supplementary information C. *Torque and temperature evolution during the second and last stages of mixture for the reference sample containing 0 phr CNT and 55 phr of silica*

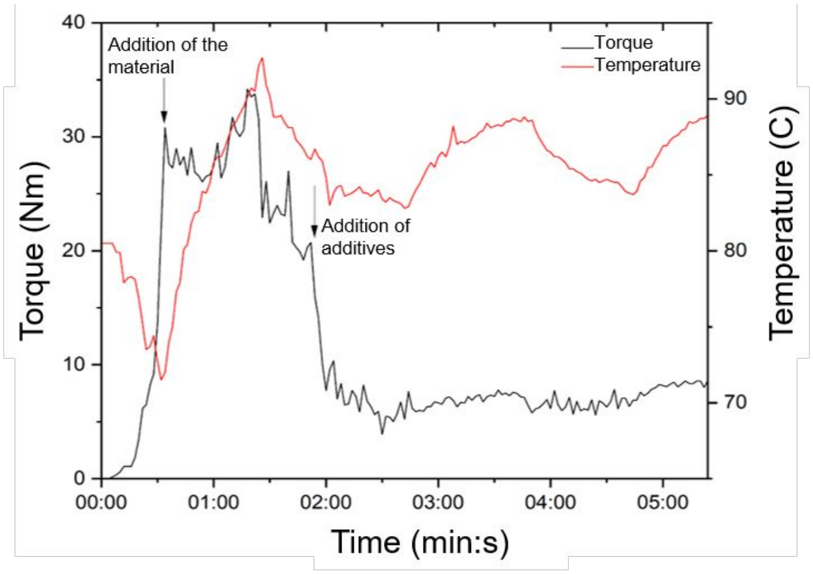

Figure S5. Torque and temperature evolution during the second stage mixture for the reference sample (55 phr silica and 0 phr CNT)

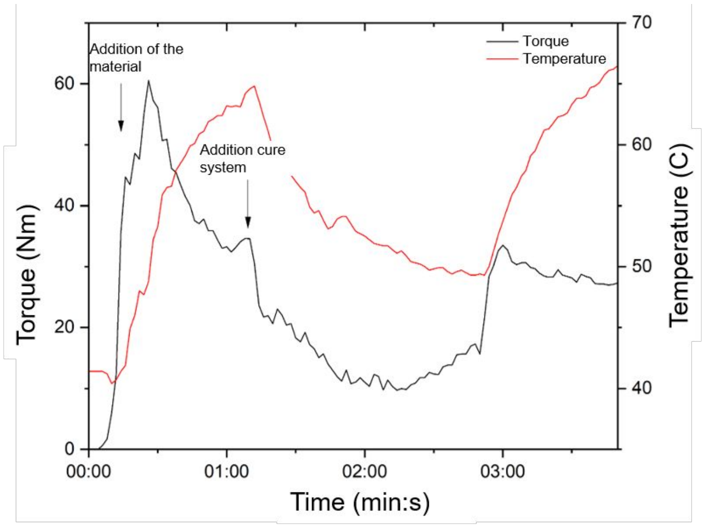

Figure S6. Torque and temperature evolution during the last stage mixture for the reference sample (55 phr silica and 0 phr CNT)
